# Data of characterization and related assays of lipid-core nanocapsule formulations and their hydrolysis mechanism

**DOI:** 10.1016/j.dib.2018.10.027

**Published:** 2018-10-12

**Authors:** Selma Calgaroto, Lucas E. Fauri, Luiza A. Frank, Karina Paese, Sílvia S. Guterres, Adriana R. Pohlmann

**Affiliations:** a Departamento de Química Orgânica and Programa de Pós-Graduação em Química, Instituto de Química, Universidade Federal do Rio Grande do Sul (UFRGS), Porto Alegre CEP 91501-970, RS, Brazil; b Programa de Pós-Graduação em Ciências Farmacêuticas, Faculdade de Farmácia, Universidade Federal do Rio Grande do Sul (UFRGS), Porto Alegre, RS, Brazil; c Departamento de Produção e Controle de Medicamentos, Universidade Federal do Rio Grande do Sul (UFRGS), Porto Alegre, RS, Brazil

## Abstract

The data presented here are related to the research paper entitled “Chemical stability, mass loss and hydrolysis mechanism of sterile and non-sterile lipid-core nanocapsules: the influence of the molar mass of the polymer wall,” [1]. Experimental details of the nanoemulsion and nanosphere preparation. Sterilization methodology and their efficacy by microbiological analyses (turbidimetry and fungi and bacteria detection). Characterization data of formulations, LNC 1, LNC 2 and LNC 3, analyzed by laser diffraction and DLS analysis, as well as, characterization data of degradation by SEC, including all statistics analyses.

**Specifications table**

**Table:** 

Subject area	*Chemistry, Pharmacy*
More specific subject area	*Hydrolysis mechanism of polymeric nanocapsules*
Type of data	*Tables, images and figures*
How data was acquired	*SEC by GPCMax tripledetector (Viscotek, Malvern Instruments Ltd, England, UK, columns of Styragel 10*^*4*^*, 10*^*5*^*, and 10*^*6*^* Å), laser diffraction (Malvern Mastersizer® 2000, Malvern Instruments, UK), dynamic light scattering (DLS, Malvern Zetasizer instrument - NanoZS, Malvern Instruments, UK) and absorbance values were measured by spectrometry (Spectramax M2e – SoftMax Pro Software Interface 5) at 370 nm.*
Data format	*Raw, analyzed*
Experimental factors	*SEC analyses were performed after extraction procedure of all constituents of LNC and separation of precipitate (polymer) and supernatant (lower molar mass constituents).*
	*Microscopy images, turbidimetry and diameter analyses of LNC were obtained without pre-treatment of samples.*
Experimental features	*Details of experimental methodologies used in this study such as preparation of nanoemulsion and nanosphere formulations. Sterilization process of LNC formulations and the efficacy of this technique by microbiological analyses. Characterization of LNC formulations after storage by SEC to identify the predominant hydrolysis mechanism. Particle size analyses to characterize physicochemical stability.*
Data source location	*Porto Alegre, Brazil*
Data accessibility	*Data is provided with this article*
Related research article	[1] S. Calgaroto, L. E. Fauri, L. Frank, K. Paese, S. S. Guterres, A. R. Pohlmann, Chemical stability, mass loss and hydrolysis mechanism of sterile and non-sterile lipid-core nanocapsules: the influence of the molar mass of the polymer wall, *Reactive and Functional Polymers, 2018*

**Value of the data**

• *SEC molar mass profiles of all the material constituents of polymeric nanocapsules is innovative for the scientific community since for most of the investigations just the polymer wall is evaluated for complex colloidal systems.*
• *Microbiological analyses of sterile and non-sterile LNC formulations provided information on the efficiency of the sterilization process and were are useful for their evaluation.*
• *Laser diffraction and dynamic light scattering (DLS) were compared with data from other works when analyzed the storage of similar delivery system and prove that physical parameters does not suffer alteration during the storage.*

## 1 Data

**Fig. 1:**
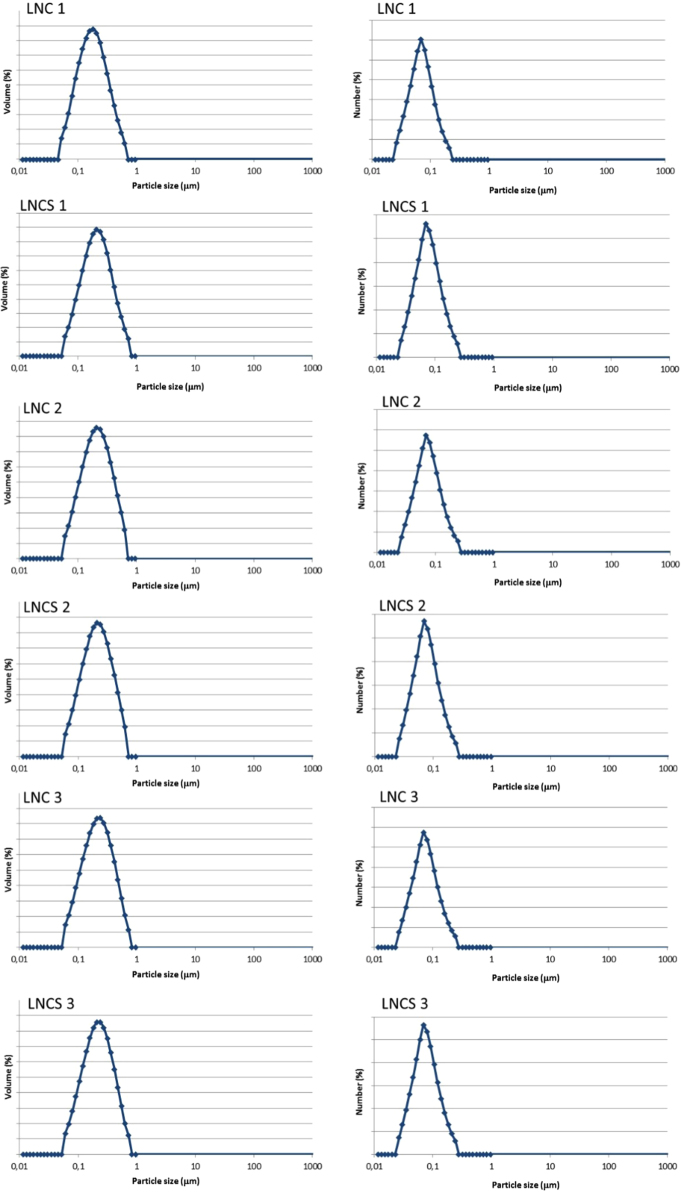
Size distribution profiles by laser diffraction of nanocapsule formulations: before (LNC) and after (LNCS) sterilization process. The data are expressed by volume of particles (left) and by number of particles (right).

**Fig. 2:**
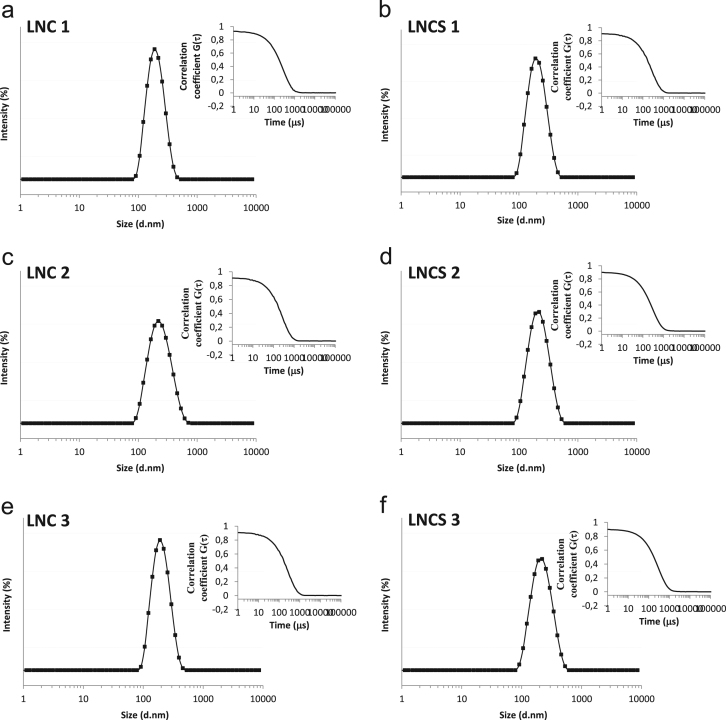
Size distribution profiles by DLS analysis of nanocapsule formulations: before (LNC) and after (LNCS) sterilization process, expressed by intensity (%). Inset: self-correlation function coefficient - G(τ) versus decay time - (μs), has been computed by applying the CONTIN method.

**Fig. 3:**
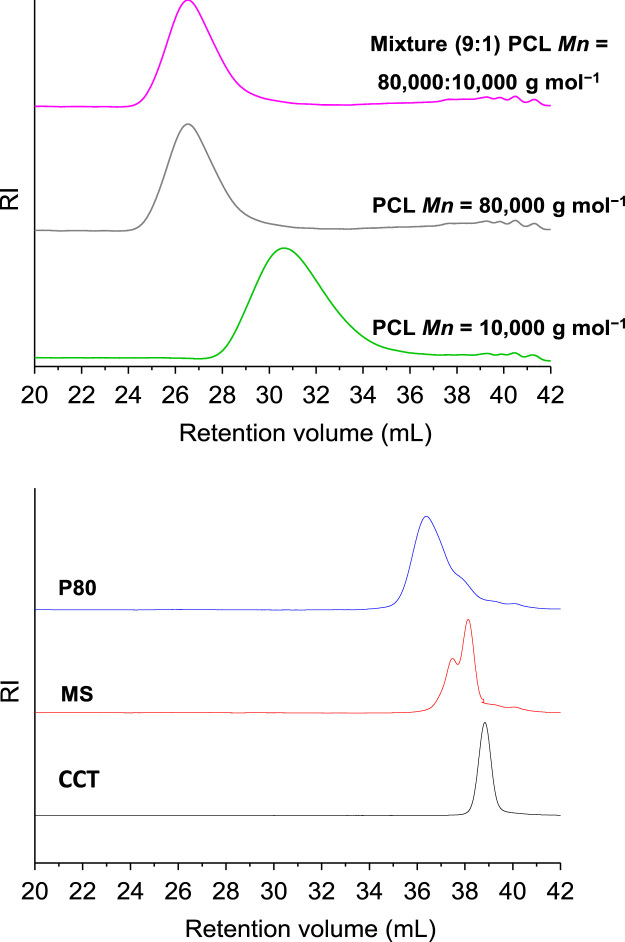
Curves of molecular weight distribution of each LNC constituent material, obtained by size exclusion chromatography (SEC).

**Fig. 4:**
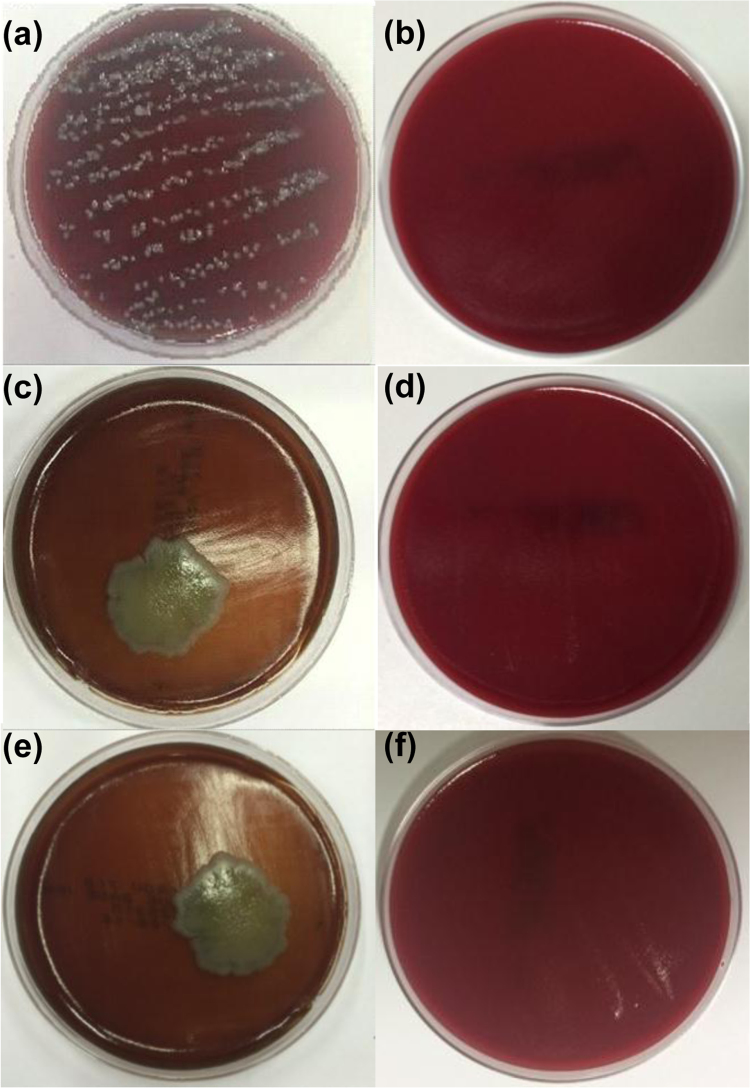
Inoculation of the non-sterile LNC 1 (a), LNC 2(c) and LNC 3 (e) and sterile formulations LNCS 1(b), LNCS 2 (d) and LNCS 3 (f) in a blood agar plate during 48 h at 37 ± 1 °C.

**Fig. 5:**
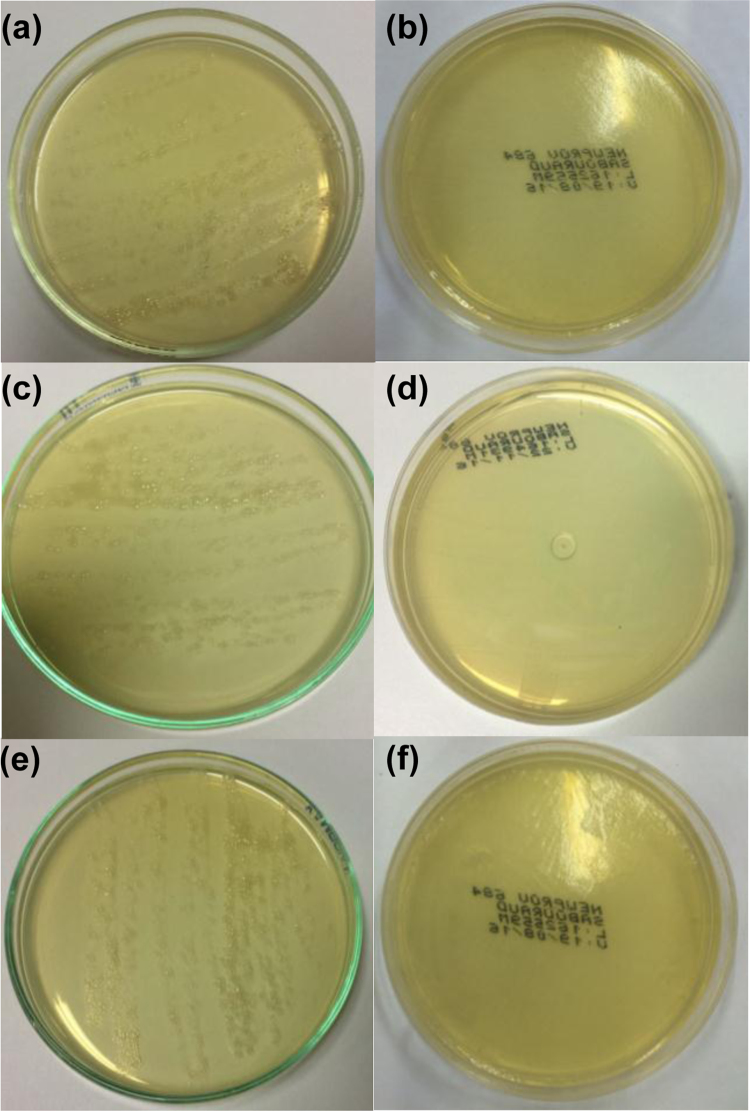
Inoculation of the non-sterile LNC 1 (a), LNC 2(c) and LNC 3 (e) and sterile formulations LNCS 1(b), LNCS 2 (d) and LNCS 3 (f) in a *Sabouraud* plate during 48 h at 35 ± 1 °C.

**Fig. 6:**
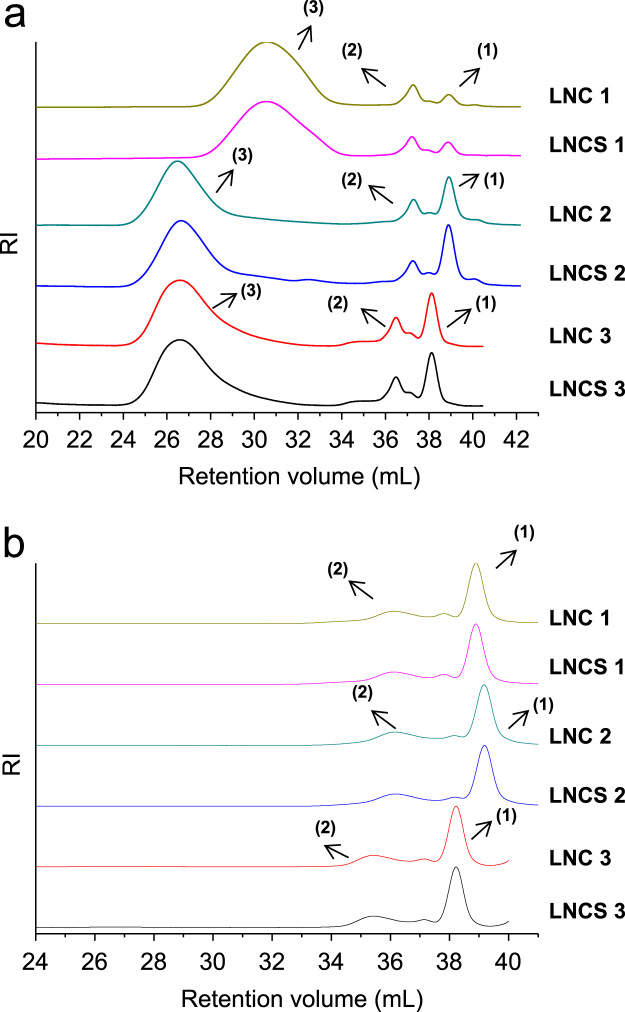
Molecular weight distribution profiles for LNCs, before (LNC) and after sterilization (LNCS): (a) precipitate and (b) supernatant, obtained by size exclusion chromatography (SEC). In (a), peaks (1), (2) and (3) are equivalent to the molecular weight of CCT, MS + P80 and PCL, respectively.

**Fig. 7:**
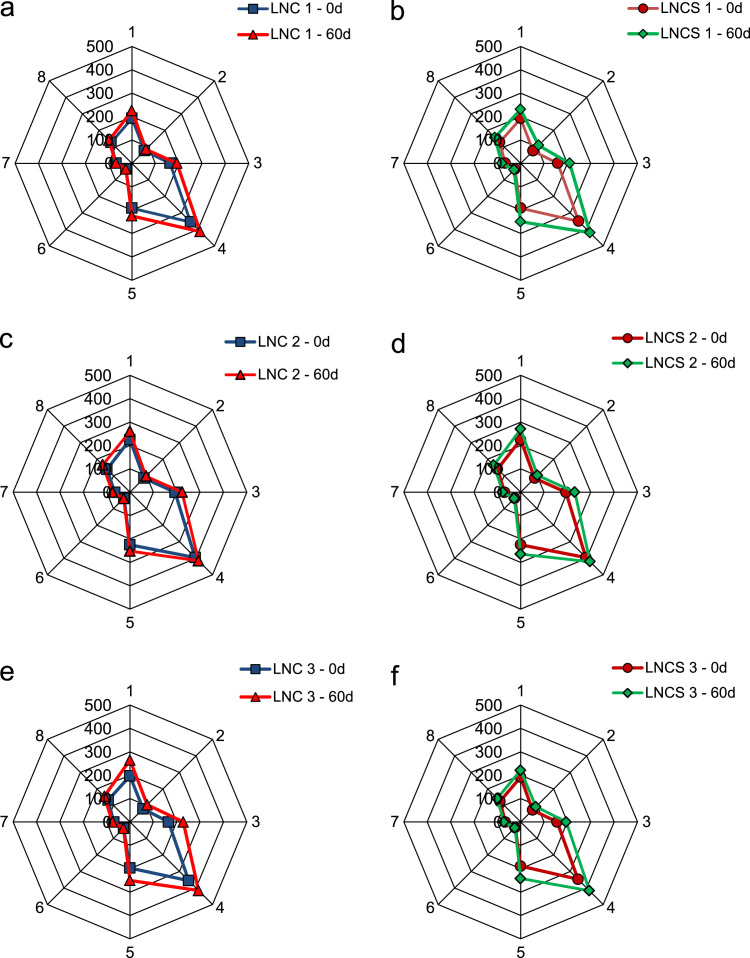
Radar charts plot for nanocapsules formulations, non-sterile (LNC) and sterile (LNCS), storage at 5 °C (60 days). [axes: 1 - volume-weighted mean diameter by volume of particles, d[3,4]v; 2 - diameter by volume at percentile 10 under the distribution curve, d(0.1)v; 3 - diameter by volume at percentile 50 under the distribution curve, d(0.5)v; 4 - diameter by volume at percentile 90 under the distribution curve, d(0.9)v; 5 - volume-weighted mean diameter by number of particles, d[3,4]n; 6 - diameter by number at percentile 10 under the distribution curve, d(0.1)n; 7 - diameter by number at percentile 50 under the distribution curve, d(0.5)n; 8 - diameter by number at percentile 90 under the distribution curve, d(0.9)n].

**Fig. 8:**
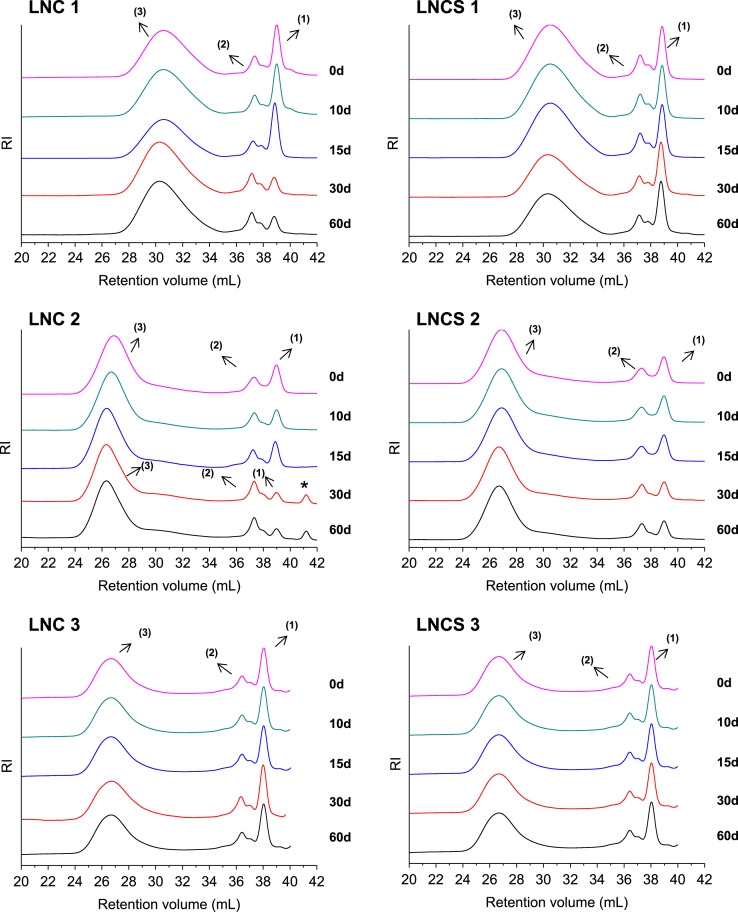
Molecular weight distribution profiles for nanocapsule formulations (precipitate), storage at 5 °C (60 days). Results obtained by size exclusion chromatography (SEC). LNC - non-sterile; LNCS – sterile. Peaks (1), (2) and (3) are equivalent to the molecular weight of CCT, MS + P80 and PCL, respectively.

**Fig. 9:**
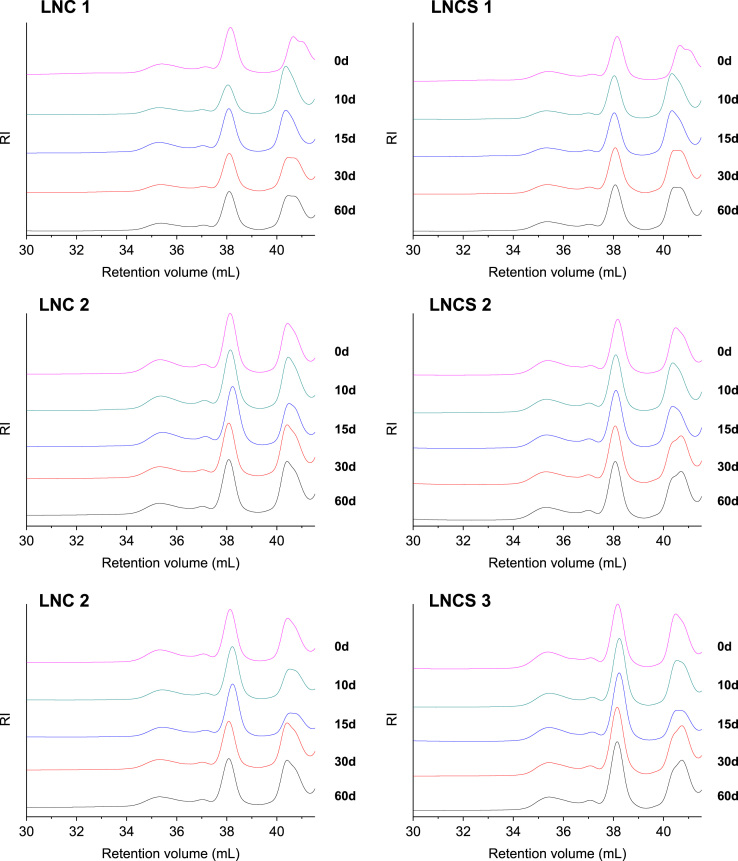
Molecular weight distribution profiles for nanocapsule formulations (supernatant) - LNC - non-sterile; LNCS – sterile, storage at 5 °C (60 days). Results obtained by size exclusion chromatography (SEC).

The data presented in Section 1.1 is the initial physicochemical characterization of LNC formulations, prior and after sterilization process. The size distribution profiles of formulations are showed in Fig. 1 and the DLS profile in Fig. 2. Section 1.2 involves the determination of each LNC constituent material by size exclusion chromatography (SEC) (Fig. 3). The data presented in Section 1.3 includes the determination of crystallinity degree for the LNC constituents and LNC formulations, prior and after sterilization process (Table 1). Section 1.4 brings data referent to sterilization process and microbiological analyses that prove their efficacy (Table 2, Fig. 4, Fig. 5). The data containing in Section 1.5 is related to the SEC profiles and molecular weight changes for the LNC constituents, prior and after sterilization (Fig. 6 and Table 3). Section 1.6 show data referent to physicochemical characterization for nanocapsules formulations, non-sterile (LNC) and sterile (LNCS), storage at 5 °C (60 days) by laser diffraction (Fig. 7) and DLS analyses (Table 4). Section 1.7 presented the changes on molar mass of nanocapsules formulations (precipitate – Fig. 8 and supernatant – Fig. 9) storage at 5 °C (60 days). The statistical analyses applied in all SEC results are presented in the same section (Table 5, Table 6, Table 7).

**Table 1:** Degree of crystallinity (%) of formulations, before (LNC) and after (LNCS) sterilization process and of the PCL with different molar masses by XRD.

Sample/material	Degree of crystallinity (%)
LNC 1	65.9
LNCS 1	65.6
LNC 2	59.2
LNCS 2	59.6
LNC 3	62.8
LNCS 3	63.4
PCL 1	83.1
PCL 2	78.9
PCL 3	64.1

PCL 1 = *M*_*n*_ 10 kg mol^-1^; PCL 2 = mixture (1:9, w/w) of PCL *M*_*n*_ 10 kg mol^-1^ and *M*_*n*_ 80 kg mol^-1^, respectively; PCL 3 = *M*_*n*_ 80 kg mol^-1^

**Table 2:** Absorbance values of the formulations after 48 h of incubation with LB Medium (λ = 370 nm). Values represent mean ± standard deviation (n=3).

**Formulation**	**1μL mL**^**-1**^	**5μL mL**^**-1**^	**10μL mL**^**-1**^
Incubated LNCS 1	0.18 ± 0.01	0.33 ± 0.00	0.56 ± 0.01
Control LNCS 1	0.19 ± 0.01	0.34 ± 0.02	0.56 ± 0.01
Incubated LNCS 2	0.21 ± 0.00	0.62 ± 0.02	0.80 ± 0.02
Control LNCS 2	0.24 ± 0.01	0.65 ± 0.01	0.80 ± 0.02
Incubated LNCS 3	0.24 ± 0.00	0.65 ± 0.02	0.83 ± 0.02
Control LNCS 3	0.23 ± 0.01	0.63 ± 0.02	0.87 ± 0.01
Incubated LNC 1	0.80 ± 0.04*	0.88 ± 0.01*	0.93 ± 0.01*
Control LNC 1	0.22 ± 0.01	0.36 ± 0.01	0.57 ± 0.02
Incubated LNC 2	0.35 ± 0.02*	0.72 ± 0.01*	0.92 ± 0.01*
Control LNC 2	0.32 ± 0.01	0.68 ± 0.01	0.86 ± 0.01
Incubated LNC 3	0.36 ± 0.02*	0.75 ± 0.01*	0.94 ± 0.01*
Control LNC 3	0.32 ± 0.02	0.69 ± 0.02	0.89 ± 0.01

* Significantly different p<0.05 compared to the respective control by the Tukey *post-hoc* test.

**Table 3:** Retention Volume (mL), number average molecular weight (*M*_*n*_), molecular weight (*M*_*w*_) and dispersity (Đ = *Mw/Mn*) of LNC (supernatant), before and after sterilization process, by size exclusion chromatography (SEC).

Sample	Peaks by SEC	Retention volume (mL)	*M*_*n*_	*M*_*w*_	Dispersity (Đ = *M*_*w*_/*M*_*n*_)
LNC 1	(1)	38.09	490	661	1.10
	(2)	37.28	2020	1779	1.08
LNCS 1	(1)	38.09	574	631	1.16
	(2)	37.28	1685	1843	1.09
LNC 2	(1)	38.09	657	723	1.10
	(2)	37.28	1653	1832	1.11
LNCS 2	(1)	38.09	688	725	1.05
	(2)	37.28	1710	1930	1,13
LNC 3	(1)	38.11	678	905	1.33
	(2)	36.50	1690	2000	1.18
LNCS 3	(1)	38.11	656	902	1.37
	(2)	36.50	1740	1980	1.14

Values represent mean ± standard deviation (n = 3) *significantly different p<0.05 (Analysis of variance (ANOVA), followed by the Tukey *post-hoc* test). Analysis performed comparing each peak, before and after sterilization process.

**Table 4:** *z*-Average diameters and polydispersity (PDI) determined by dynamic light scattering (DLS) for formulations before and after the storage time (60 days) (semi-dilute regimen).

Formulation	Storage time (days)	PCS (Method of Cumulants)	
		*z-average* diameters (nm)	PDI (dimensionless)
LNC 1	0	198 ± 10	0.10 ± 0.03
	60	213 ± 4	0.11 ± 0.02
LNCS 1	0	200± 3	0.11 ± 0.04
	60	205± 16	0.10 ± 0.03
LNC 2	0	207 ± 9	0.10 ± 0.03
	60	211 ± 3	0.11 ± 0.01
LNCS 2	0	202 ± 5	0.13 ± 0.01
	60	218 ± 2	0.13 ± 0.03
LNC 3	0	202 ± 9	0.12 ± 0.01
	60	211 ± 14	0.11 ± 0.02
LNCS 3	0	185 ± 2	0.09 ± 0.00
	60	184 ± 3	0.10 ± 0.01

Values represent mean ± standard deviation (n = 3) *significantly different p<0.05 (Analysis of variance (ANOVA), followed by the Tukey *post-hoc* test). Analysis performed comparing each sample, before and after 60 days of storage time.

**Table 5:** Statistical analysis of the weight loss [∆*M*_*w*_ (%)] and dispersity (Đ = *M*_*w*_*/M*_*n*_) for the non-sterile and sterile nanocapsule formulations (precipitate) under storage, at 5 °C, by SEC. Peaks (1), (2) and (3) are equivalent to the molecular weight of CCT, MS + P80 and PCL, respectively.

Storage (days)	Peak (SEC)	Weight loss (∆*M*_*w*_ %)						Dispersity (Đ = *M*_*w*_/*M*_*n*_)					
		LNC 1	LNCS 1	LNC 2	LNCS 2	LNC 3	LNCS 3	LNC 1	LNCS 1	LNC 2	LNCS 2	LNC 3	LNCS 3
0	(1)	^A^0^a^	^A^0^a^	^A^0^a^	^A^0^a^	^A^0^a^	^A^0^a^	^A^1.20^a^	^A^1.17^a^	^A^1.18^a^	^A^1.27^a^	^A^1.08^a^	^A^1.08^a^
	(2)	^A^0^a^	^A^0^a^	^A^0^a^	^A^0^a^	^A^0^a^	^A^0^a^	^A^1.17^a^	^A^1.17^a^	^A^1.23^a^	^A^1.26^a^	^A^1.22^a^	^A^1.22^a^
	(3)	^A^0^a^	^A^0^a^	^A^0^a^	^A^0^a^	^A^0^a^	^A^0^a^	^A^1.47^a^	^A^1.47^a^	^A^2.29^a^	^A^2.28^a^	^A^2.25^a^	^A^2.27^a^
10	(1)	^A^0^a^	^A^0^a^	^A^1.2^a^	^A^1.5^a^	^A^1.4^a^	^A^2.5^a^	^A^1.17^a^	^A^1.18^a^	^A^1.19^a^	^A^1.21^a^	^A^1.07^a^	^A^1.22^b^
	(2)	^A^0^a^	^A^0^a^	^A^0.8^a^	^A^0.9^a^	^A^7.6^a^	^A^8.5^a^	^A^1.16^a^	^A^1.20^a^	^A^1.20^a^	^A^1.20^a^	^A^1.15^a^	^A^1.18^a^
	(3)	^A^0^a^	^A^0^a^	^A^13.6^b^	^B^3.5^a^	^A^16.0^b^	^B^5.0^a^	^A^1.54^a^	^A^1.52^a^	^A^2.21^b^	^A^2.29^a^	^A^2.18^a^	^A^2.27^a^
15	(1)	^A^7.4^a^	^A^0.2^a^	^A^3.9^a^	^A^4.4^a^	^A^14.1^b^	^B^2.4^a^	^A^1.27^a^	^B^1.16^a^	^A^1.20^a^	^A^1.20^a^	^A^1.12^a^	^A^1.10^a^
	(2)	^A^0^a^	^A^0^a^	^A^6.5^a^	^A^8.1^a^	^A^6.5^a^	^A^7.1^a^	^A^1.17^a^	^A^1.23^a^	^A^1.21^a^	^A^1.15^a^	^A^1.16^a^	^A^1.16^a^
	(3)	^A^3.8^a^	^A^3.2^a^	^A^13.8^b^	^B^2.8^a^	^A^17.2^b^	^B^8.2^a^	^A^1.58^a^	^A^1.51^a^	^A^2.37^b^	^A^2.31^a^	^A^2.15^a^	^A^2.25^a^
30	(1)	^A^40.7^b^	^B^18.4^b^	^A^46.7^b^	^B^18.2^b^	^A^18.6^b^	^B^5.1^a^	^A^1.13^a^	^A^1.10^a^	^A^1.41^b^	^B^1.15^a^	^A^1.23^b^	^A^1.15^a^
	(2)	^A^41.4^b^	^B^1.3^a^	^A^35.1^b^	^B^14.9^b^	^A^4.1^a^	^A^5.2^a^	^A^1.12^a^	^A^1.15^a^	^A^1.29^a^	^B^1.40^b^	^A^1.16^a^	^A^1.11^b^
	(3)	^A^29.3^b^	^B^18.3^b^	^A^28.5^c^	^B^15.0^b^	^A^24.3^b^	^B^12.5^b^	^A^1.78^b^	^B^1.50^a^	^A^2.46^b^	^B^2.29^a^	^A^2.07^b^	^B^2.23^a^
60	(1)	^A^54.2^c^	^B^21.3^c^	^A^50.0^b^	^B^33.8^c^	^A^20.4^b^	^B^1.0^a^	^A^1.33^b^	^A^1.35^b^	^A^1.61^b^	^B^1.35^b^	^A^1.23^b^	^A^1.27^b^
	(2)	^A^46.1^b^	^B^13.7^b^	^A^35.7^b^	^B^25.5^c^	^A^6.9^a^	^A^7.9^a^	^A^1.05^a^	^B^1.20^a^	^A^1.32^b^	^A^1.35^b^	^A^1.13^b^	^A^1.13^b^
	(3)	^A^43.0^c^	^B^28.9^c^	^A^31.7^c^	^B^19.8^b^	^A^28.4^b^	^B^18.2^b^	^A^1.55^a^	^A^1.52^a^	^A^2.47^b^	^A^2.41^b^	^A^2.01^b^	^B^2.14^b^

^a,b,c^ analysis performed by row between the different storage times for each independent formulation;

^A,B^ analysis performed by lines comparing the "sterile and non-sterile" condition for each storage time;

Equivalent letters means statistical equivalence (p >0.05). Bonferroni׳s test was used as post test. Values are expressed as average (n=3).

**Table 6:** Statistical analysis of the number average molecular weight (*M*_*n*_) and molecular weight (*M*_*w*_) for the non-sterile and sterile nanocapsule formulations (precipitate), under storage, at 5 °C, by SEC. Peaks (1), (2) and (3) are equivalent to the molecular weight of CCT, MS + P80 and PCL, respectively.

Storage (days)	Peak (SEC)	*M*_*n*_						*M*_*w*_					
		LNC 1	LNCS 1	LNC 2	LNCS 2	LNC 3	LNCS 3	LNC 1	LNCS 1	LNC 2	LNCS 2	LNC 3	LNCS 3
0	(1)	^A^1024^a^	^A^1,031^a^	^A^1047^a^	^A^1045^a^	^A^652^a^	^A^655^a^	^A^1228^a^	^A^1203^a^	^A^1231^a^	^A^1320^a^	^A^706^a^	^A^710^a^
	(2)	^A^2777^a^	^A^2799^a^	^A^3010^a^	^A^3065^a^	^A^1799^a^	^A^1798^a^	^A^3240^a^	^A^3280^a^	^A^3703^a^	^A^3870^a^	^A^2187^a^	^A^2193^a^
	(3)	^A^32,987^a^	^A^32,478^a^	^A^75,300^a^	^A^75,658^a^	^A^71,000^a^	^A^70,200^a^	^A^48,475^a^	^A^47,778^a^	^A^173,681^a^	^A^185,076^a^	^A^159,790^a^	^A^159,300^a^
10	(1)	^A^1019^a^	^A^1,014^a^	^A^1024^a^	^A^1045^a^	^A^649^a^	^A^579^a^	^A^1195^a^	^A^1200^a^	^A^1216^a^	^A^1265^a^	^A^695^a^	^A^680^a^
	(2)	^A^2769^a^	^A^2798^a^	^A^3015^a^	^A^3042^a^	^A^1757^a^	^A^1750^a^	^A^3220^a^	^A^3300^a^	^A^3646^a^	^A^3670^a^	^A^2020^a^	^A^2075^a^
	(3)	^A^31,377^a^	^A^31,338^a^	^A^69,600^a^	^A^75,505^a^	^A^60,900^b^	^A^67,000^b^	^A^48,400^a^	^A^47,545^a^	^A^161,379^b^	^B^178,330^a^	^A^132,875^b^	^B^152,280^a^
15	(1)	^A^896^b^	^B^1,026^a^	^A^990^b^	^A^1044^a^	^A^542^b^	^A^624^a^	^A^1138^a^	^A^1200^a^	^A^1185^a^	^A^1257^a^	^A^605^b^	^A^689^a^
	(2)	^A^2760^a^	^A^2795^a^	^A^2887^a^	^A^3033^a^	^A^1764^a^	^A^1748^a^	^A^3238^a^	^A^3500^a^	^A^3489^a^	^A^3411^a^	^A^2044^a^	^A^2031^a^
	(3)	^A^29,453^a^	^A^30,590^a^	^A^67,030^b^	^B^75,515^a^	^A^60,213^b^	^A^65,000^b^	^A^46,615^a^	^A^46,550^a^	^A^161,000^b^	^B^180,280^a^	^A^129,600^b^	^B^146,178^a^
30	(1)	^A^647^b,c^	^B^907^b^	^A^463^c^	^B^955^a^	^A^467^b^	^A^585^a^	^A^729^b^	^B^983^b^	^A^655^b^	^B^1080^a^	^A^575^b^	^A^672^a^
	(2)	^A^1687^b^	^B^2082^b^	^A^1860^b^	^B^2400^b^	^A^1820^a^	^A^1940^a^	^A^1898^b^	^B^3240^a^	^A^2405^b^	^B^3290^a^	^A^2105^a^	^A^2145^a^
	(3)	^A^19,278^b^	^B^25,935^b^	^A^54,300^c^	^B^68,550^b^	^A^57,952^b^	^A^62,490^b^	^A^34,068^b^	^B^38,995^b^	^A^133,608^c^	^B^157,300^b^	^A^119,870^c^	^B^139,350^a^
60	(1)	^A^427^c^	^B^573^b^	^A^386^c^	^B^617^b^	^A^467^b^	^A^553^a^	^A^562^c^	^B^946^b^	^A^615^b^	^B^835^b^	^A^573^b^	^B^700^a^
	(2)	^A^1659^b^	^B^2010^b^	^A^1838^b^	^A^2020^b^	^A^1765^a^	^A^1839^a^	^A^1748^b^	^B^2830^b^	^A^2380^b^	^A^2729^b^	^A^2000^a^	^A^2085^a^
	(3)	^A^18,140^b^	^B^22,450^b^	^A^52,415^c^	^B^59,350^b^	^A^56,850^b^	^A^61,000^b^	^A^27,615^c^	^B^33,975^b^	^A^127,500^c^	^B^143,000^b^	^A^114,500^c^	^B^130,500^b^

^a,b,c^ analysis performed by row between the different storage times for each peak of the each independent formulation;

^A,B^ analysis performed by lines comparing the "sterile and non-sterile" condition for each storage time;

Equivalent letters means statistical equivalence (p >0.05). Bonferroni׳s test was used as post test. Values are expressed as average (n=3).

**Table 7:** Statistical analysis of the number average molecular weight (*M*_*n*_) and molecular weight (*M*_*w*_) for the non-sterile and sterile nanocapsule formulations (supernatant), under storage, at 5 °C, by SEC.

Storage (days)	*M*_*n*_						*M*_*w*_					
	LNC 1	LNCS 1	LNC 2	LNCS 2	LNC 3	LNCS 3	LNC 1	LNCS 1	LNC 2	LNCS 2	LNC 3	LNCS 3
0	^A^236^a^	^A^244^a^	^A^320^a^	^A^320^a^	^A^325^a^	^A^295^a^	^A^1027^a^	^A^1020^a^	^A^1126^a^	^A^1199^a^	^A^1087^a^	^A^1030^a^
10	^A^268^a^	^A^235^a^	^A^269^a^	^A^287^a^	^A^264^a^	^A^283^a^	^A^996^a^	^A^997^a^	^A^1047^a^	^A^1126^a^	^A^983^a^	^A^944^a^
15	^A^310^a^	^A^270^a^	^A^370^a^	^A^290^a^	^A^262^a^	^A^255^a^	^A^1025^a^	^A^1016^a^	^A^1059^a^	^A^1128^a^	^A^976^a^	^A^949^a^
30	^A^264^a^	^A^277^a^	^A^270^a^	^A^266^a^	^A^252^a^	^A^264^a^	^A^897^b^	^A^929^a^	^A^1087^a^	^A^1135^a^	^A^923^a^	^A^882^b^
60	^A^126^b^	^A^125^b^	^A^135^b^	^A^146^b^	^A^173^b^	^A^175^b^	^A^430^c^	^A^420^b^	^A^450^b^	^A^491^b^	^A^684^b^	^B^773^b^

^a,b,c^ analysis performed by row between the different storage times for each independent formulation;

^A,B^ analysis performed by lines comparing the "sterile and non-sterile" condition for each storage time;

Equivalent letters means statistical equivalence (p>0.05). Bonferroni׳s test was used as post test. Values are expressed as average (n=3).

### 1.1 Initial physicochemical characterization of LNC formulations, prior and after sterilization process

 See Fig. 1, Fig. 2.

### 1.2 Size exclusion chromatography (SEC) analysis of each LNC constituent material

 See Fig. 3.

### 1.3 Crystallinity measurement for the LNC constituents and LNC formulations, prior and after sterilization process

 See Table 1.

### 1.4 Microbiological contamination assays

#### 1.4.1 Turbidimetry test

 See Table 2.

#### 1.4.2 Fungi and bacteria detection

 See Fig. 4, Fig. 5.

### 1.5 Molecular weight changes for the LNC constituents, prior and after sterilization process

 See Fig. 6 and Table 3.

### 1.6 Physicochemical characterization for nanocapsule formulations, non-sterile (LNC) and sterile (LNCS), storage at 5 °C (60 days)

#### 1.6.1 Particle size analyses by laser diffraction – radar charts

 See Fig. 7 and Table 4.

### 1.7 Molecular weight distribution profiles for nanocapsules formulations (precipitate and supernatant) storage at 5 °C (60 days)

 See Fig. 8, Fig. 9; Table 5, Table 6, Table 7.

## 2 Experimental design, materials and methods

The methodologies to obtain the data exposed here are described in Calgaroto et al. [1].

### 2.1 Preparation of Nanoemulsion (NE) and Nanosphere (NS) formulations

To prepare NE formulation, an organic phase containing 0.038 g of sorbitan monostearate, 0.160 g of oil (capric/caprylic triglyceride) and 27 mL acetone was injected into an aqueous phase containing 0.080 g polysorbate 80 and 53 mL ultrapure water, under magnetic stirring at 40 °C. To prepare NS, the organic phase was composed by 0.100 g poly(ε-caprolactone), 0.038 g of sorbitan monostearate solubilized in 27 mL acetone. This phase was injected into an aqueous phase containing 0.080 g polysorbate 80 and 53 mL ultrapure water, under magnetic stirring at 40 °C. For both formulations, after 10 min, the organic solvent was removed under reduced pressure at 40 °C using a rotary evaporator (Büchi, Switzerland), having their volume reduced to 10 mL. The formulations prepared in triplicate batches (n = 3).

### 2.2 Sterilization process

Sterilization was performed in Horizontal Autoclave (Phoenix AB42, São Paulo, Brazil) at 134 °C for 10 min and 2.10 bar as previously described [2]. Initially, 5 mL of each formulation was packed in ampoule bottles (h = 100 mm; ᴓ = 5 mm; v = 10 mL) using a micropipette (#BR704764) (BRAND® Transferpette® S pipette, single channel) acquired from Sigma-Aldrich (Steinheim, Germany). This container were sealed with rubber stoppers and aluminum seals with the aid of a climper (#224321) purchased from SKS Science Products (Watervliet, New York). The ampoule bottles, rubber stoppers and aluminum seals were purchase from Galia Embalagens (Porto Alegre, Brazil). After sterilization, the formulations were immediately removed from the autoclave and kept at 5 °C in a refrigerator (model: CRM33, Consul®, Brazil), under light.

### 2.3 Particle size analyses

The nanocapsule formulations were evaluated (particle size distribution) by laser diffraction (LD) using a Malvern Mastersizer® 2000 instrument (Malvern Instruments, UK). The sample (n=3) was placed in the equipment using a micropipette (#Z646598) (BRAND® Transferpette® S pipette, single channel) acquired from Sigma-Aldrich (Steinheim, Germany) device wet unit (Hydro 2000SM – AWM2002 – Malvern, UK) in an amount sufficient to obtain more than 2% obscuration. Mie theory of light scattering was used to calculate the particle size distribution. Mean diameter was expressed as volume-weighted mean diameter (d4,3), and polydispersity (Span) was calculated using Eq. (1), where d0.9, d0.1, and d0.5 are respectively the diameters at percentiles 90, 10, and 50 of the cumulative size distribution curve. The median diameter by number of particles (d0.5)n was also determined for each sample using the distribution curve based on the number of particles.

(1) $$\mathrm{Span}=\left(\frac{d_{0.9}-d_{0.1}}{d_{0.5}}\right)$$

Dynamic light scattering (DLS) was carried out to determine the mean hydrodynamic diameter and the polydispersity of the submicrometric particle populations in a Nanoseries® ZetaSizer ZS (Malvern, UK) equipment. LNC formulations (20 μL) were diluted in MilliQ® water (10 mL) previously filtered (0.45 μm, hydrophilic membrane (#HVLP) (Durapore®, Merck, Germany). Each sample was poured into a quartz flow cell (#ZEN0023, Malvern, UK). The scattered light was detected at an angle of 173°. The correlograms were fit using the method of Cumulants to calculate the z-average diameters. Experiments were conducted with three batches for each sample.

## 3 Microbiological contamination assays

### 3.1 Turbidimetry test

The microbiological contamination was evaluated by a turbidimetry method to identify the presence of microorganisms in the formulation prior (LNC) and after sterilization process (LNCS). The presence of contaminants in the sample is related with the increase in absorbance [3]. The absorbance of formulations without incubation (LNC and LNCS) was determined as the controls. The formulations (LNC and LNCS) were incubated at 37 ± 1 °C during 48 h using three different concentrations by adding 1, 5 or 10 μL of each sample into 1 mL of Luria Bertani (LB) medium. The experiments were performed in triplicate, and the absorbance values were measured by spectrometry (Spectramax M2e – SoftMax Pro Software Interface 5) at 370 nm.

### 3.2 Fungi and bacteria detection

The detection of fungi and bacteria in the formulations was performed by inoculating 20 μL of each formulation (LNC and LNCS) for 48 h at 37 ± 1 and 35 ± 1 °C, respectively, a blood agar plate for the bacterial growing and in a *Sabouraud* plate for the fungal growing.
