# Supplementary figures and images for: Data of characterization and related assays of lipid-core nanocapsule formulations and their hydrolysis mechanism
Source: Data Brief. 2018 Oct 12;21:918–33. doi: 10.1016/j.dib.2018.10.027 (PMC6222288; doi:10.1016/j.dib.2018.10.027)

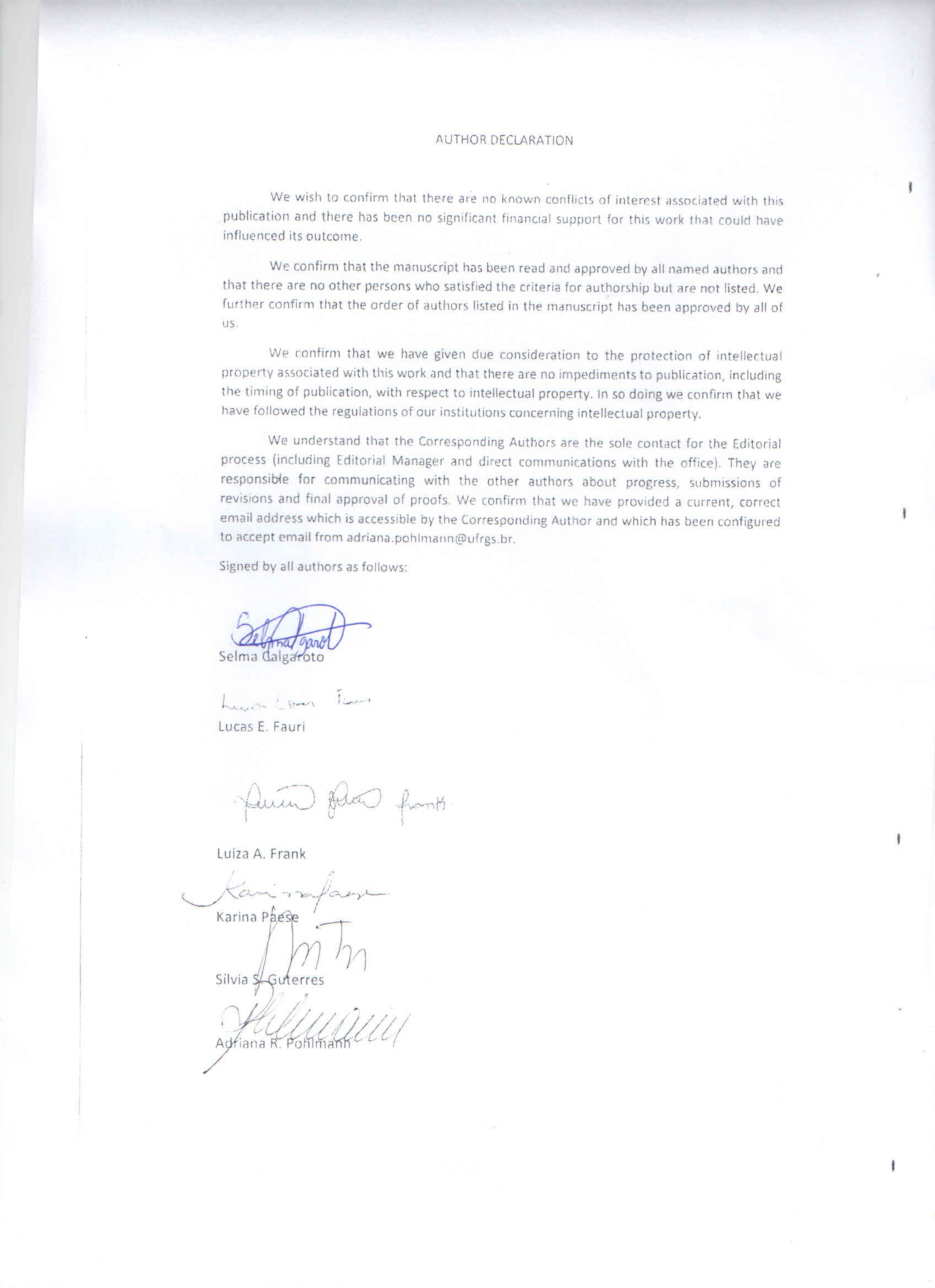

Supplement: Supplementary file 1 — Supplementary material [file mmc1.jpg]
